# MicroRNA-92a–CPEB3 axis protects neurons against inflammatory neurodegeneration

**DOI:** 10.1126/sciadv.adi6855

**Published:** 2023-11-24

**Authors:** Iris Winkler, Jan Broder Engler, Vanessa Vieira, Simone Bauer, Yi-Hsiang Liu, Giovanni Di Liberto, Katarzyna M. Grochowska, Ingrid Wagner, Jasmina Bier, Lukas C. Bal, Nicola Rothammer, Nina Meurs, Kristof Egervari, Benjamin Schattling, Gabriela Salinas, Michael R. Kreutz, Yi-Shuian Huang, Ole Pless, Doron Merkler, Manuel A. Friese

**Affiliations:** ^1^ Institute of Neuroimmunology and Multiple Sclerosis, University Medical Center Hamburg-Eppendorf, Hamburg 20251, Germany.; ^2^ Institute of Biomedical Sciences, Academia Sinica, Taipei 11529, Taiwan.; ^3^ Department of Pathology and Immunology, Division of Clinical Pathology, Geneva Faculty of Medicine, University of Geneva and University Hospital of Geneva, Geneva 1211, Switzerland.; ^4^ Leibniz Group ‘Dendritic Organelles and Synaptic Function’, Center for Molecular Neurobiology Hamburg (ZMNH), University Medical Center Hamburg-Eppendorf, Hamburg 20251, Germany.; ^5^ Research Group Neuroplasticity, Leibniz Institute for Neurobiology, Magdeburg 39118, Germany.; ^6^ Institut of Human Genetics, NGS Integrative Genomics, University Medical Center Göttingen, Göttingen 37077, Germany.; ^7^ Fraunhofer Institute for Translational Medicine and Pharmacology ITMP, Hamburg 22525, Germany.

## Abstract

Neuroinflammation causes neuronal injury in multiple sclerosis (MS) and other neurological diseases. MicroRNAs (miRNAs) are important modulators of neuronal stress responses, but knowledge about their contribution to neuronal protection or damage during inflammation is limited. Here, we constructed a regulatory miRNA–mRNA network of inflamed motor neurons by leveraging cell type–specific miRNA and mRNA sequencing of mice undergoing experimental autoimmune encephalomyelitis (EAE). We found robust induction of miR-92a in inflamed spinal cord neurons and identified cytoplasmic polyadenylation element-binding protein 3 (*Cpeb3*) as a key target of miR-92a–mediated posttranscriptional silencing. We detected CPEB3 repression in inflamed neurons in murine EAE and human MS. Moreover, both miR-92a delivery and *Cpeb3* deletion protected neuronal cultures against excitotoxicity. Supporting a detrimental effect of *Cpeb3* in vivo, neuron-specific deletion in conditional *Cpeb3* knockout animals led to reduced inflammation-induced clinical disability in EAE. Together, we identified a neuroprotective miR-92a–*Cpeb3* axis in neuroinflammation that might serve as potential treatment target to limit inflammation-induced neuronal damage.

## Teaser

By constructing miRNA–mRNA networks in neurons a miR-92a–Cpeb3 axis was discovered that protects from neuroinflammation.

## INTRODUCTION

Multiple sclerosis (MS) is the most common inflammatory disease of the central nervous system (CNS) that usually starts in young adulthood and is currently incurable (*1*, *2*). Worldwide, more than 2.5 million people are afflicted with MS, which is characterized by a wide spectrum of neurological symptoms (*1*, *3*). MS is considered to be initiated by aberrantly activated T cells that attack self-antigens of the CNS and set up chronic neuroinflammation, which eventually leads to demyelination, synaptic and neuroaxonal injury, and loss (*4*). Neurodegeneration best correlates with clinical disability in MS patients and can already be detected at early disease stages (*5*, *6*). Since currently available drugs insufficiently mitigate progression of neurodegeneration independent of the MS subtype (*2*), there is an urgent unmet clinical need for neuroprotective interventions. Therefore, it is critical to uncover neuron-intrinsic mechanisms of protection against neurodegeneration in MS to be able to tailor neuroprotective drugs that eventually halt neurological disease progression.

Recently, single-cell transcriptomic analysis of MS brains (*7*) and neuron-specific transcriptional profiling in experimental autoimmune encephalomyelitis (EAE) (*8*), a widely used animal model of MS, revealed pathways that contribute to MS pathology and are deregulated on a transcriptional level in neurons. As we have recently demonstrated for the presynaptic protein bassoon (*8*) and the epigenetic regulator G9a (*9*), neuron-specific transcriptional profiling in EAE led to the identification of factors that modulate neurodegeneration and can be therapeutically targeted. Both studies revealed substantial transcriptional dysregulation during inflammation, which in part appears to be driven by epigenetic changes. Besides posttranslational histone modifications (*8*), microRNAs (miRNAs) represent another important regulatory layer that shapes transcriptional programs by specific silencing of target mRNAs (*10*). These ~22-nucleotide short evolutionary conserved RNAs are encoded in inter- or intragenic regions, often within polycistronic clusters, and are processed by a complex biogenesis machinery (*11*). In the cytoplasm, mature miRNAs are loaded into one of the four argonaute (AGO) protein family members 1 to 4, which then assemble with other proteins to build the RNA-induced silencing complex. Within this complex, miRNAs bind complementary sites in the 3′ untranslated region (UTR) of their target mRNAs, leading to inhibition of translation or activation of mRNA decay (*11*). The importance of miRNAs is illustrated by experimental ablation of components of miRNA biogenesis or specific deletion of miRNA genes that affect the development, differentiation, and survival of neurons (*12*, *13*). Besides their role in tuning transcriptional programs in organismal and cellular development, miRNAs are important modulators of gene regulation in plasticity and maintenance of neuronal networks in adulthood (*14*). Of all protein coding genes, more than 60% are predicted to be regulated by miRNAs, highlighting the potential broad impact of these transcriptional regulators (*11*).

Here, we set out to systematically investigate neuronal miRNA profiles and their downstream effects on translated mRNAs in the EAE mouse model of MS to gain insights into neuronal resistance pathways that could be therapeutically exploited. We found a protective role of neuronal miR-92a, while its downstream target cytoplasmic polyadenylation element-binding protein 3 (*Cpeb3*) promoted neuronal demise. Accordingly, neuron-specific deletion of *Cpeb3* ameliorated clinical severity during neuroinflammation and improved neuronal resistance against excitotoxicity. We detected decreased *Cpeb3* levels in both murine EAE spinal cords and in human MS brain tissue, suggesting a consistent activation of the miR-92a–CPEB3 axis during neuroinflammation across species. Thus, our findings identify CPEB3 repression to confer neuronal resistance to neuroinflammation and open an avenue for the development of neuroprotective treatments in MS, and potentially other CNS disorders associated with inflammation.

## RESULTS

### miRNA network regulates the neuronal translatome during neuroinflammation

**Fig. 1.**
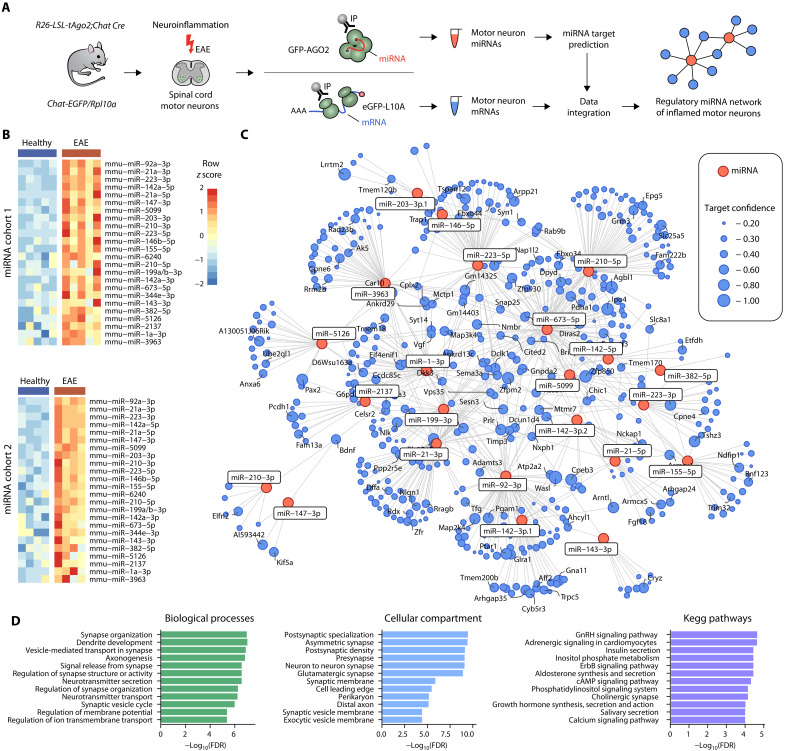
miRNA network regulates the neuronal translatome during neuroinflammation. (**A**) Acute EAE (days 12 and 15 after immunization) spinal cords were subjected to GFP-AGO2– and GFP-RPL10A–directed IP to profile motor neuron–specific miRNA and mRNA, respectively. Target prediction and data integration resulted in a miRNA–mRNA network of inflamed motor neurons. (**B**) Heatmaps of motor neuronal miRNA candidates identified across two independent cohorts (miRNA cohort 1: *n* = 5 per group; miRNA cohort 2: *n* = 4 per group). Each sample consists of three pooled cervical spinal cords. (**C**) miRNA–mRNA network with candidate miRNAs (red nodes) and their predicted mRNA targets (blue nodes; TargetScanMouse 8.0). Only targets with significant down-regulation on mRNA level were retained (*n* = 4 versus 5). Node size reflects target confidence assessed by CWCS. (**D**) Gene ontology and KEGG pathway overrepresentation analysis of identified mRNA targets with resulting terms ordered by statistical significance [false discovery rate (FDR)].

The EAE mouse model of MS features neuroinflammation in the spinal cord, accompanied by clinical motor disability that correlates with neuronal damage (*15*). To investigate the contribution of miRNAs in regulating the neuronal translatome during neuroinflammation, we performed cell type–specific miRNA and mRNA profiling of spinal cord motor neurons (Fig. 1A). For miRNA profiling, we crossed *R26-LSL-tAgo2* mice (*16*), containing a floxed stop sequence before a *GFP*-*Myc-Ago2* cassette, with mice that express Cre recombinase under control of the motor neuron–specific promotor choline acetyltransferase (ChAT). This resulted in a specific expression of GFP-MYC-AGO2 in spinal cord motor neurons but not in other spinal cord resident cells (fig. S1, A and B), allowing for immunoprecipitation (IP) of motor neuronal AGO2 with attached miRNA. By using quantitative reverse transcription polymerase chain reaction (qRT-PCR), we compared the relative enrichment of cell type–specific miRNAs after AGO2 IP to the whole spinal cord lysate to test the specificity and sensitivity of the method (*16*). Motor neuronal miR-218 and neuronal miR-138 were similarly enriched by IP in healthy and EAE mice at day 12 after immunization, while miR-9 and miR-150, broadly expressed in the nervous and hematopoietic system, respectively, were not enriched (fig. S1C). To focus on robust miRNA expression changes during neuroinflammation, we sampled and analyzed two independent cohorts (miRNA cohort 1 and cohort 2) of mice during acute EAE (day 12 after immunization) in comparison to healthy littermate controls (Fig. 1B and fig. S1D). To increase the robustness and ensure sufficient miRNA yields after IP, we pooled three mice per biological replicate. We found 24 miRNAs to be consistently deregulated in both cohorts and ranked miRNA candidates by statistical significance across cohorts.

To assess the action of miRNAs on their predicted target mRNAs, we leveraged motor neuron–specific purification of mRNAs (*8*) using *Chat-EGFP/Rpl10a* (*17*) transgenic mice that express green fluorescent protein (GFP)–tagged ribosomes under control of the *Chat* promotor. In both EAE mice (day 15 after immunization) and healthy littermates, we observed a robust 10-fold enrichment of *Chat* transcripts after IP of GFP-tagged ribosomes in comparison to whole spinal cord lysate. Other cell type–specific gene transcripts for oligodendrocytes (*Cnp*), astrocytes (*Gfap*), or immune cells (*Ptprc*) were at most marginally enriched, indicating a high specificity and sensitivity of the method (fig. S1E). Analogously to our miRNA profiling, we sampled mice during acute EAE (day 15 after immunization) in comparison to healthy littermate controls and pooled three mice per biological replicate (fig. S1F). Since we were interested in transcripts that were effectively silenced by miRNA, we focused our analysis on down-regulated mRNAs that were predicted targets of the 24 miRNA candidates. We only considered moderate- and high-confidence targets, defined by a cumulative weighted context score (CWCS) (*18*) lower than −0.2. Next, we constructed a miRNA–mRNA network of motor neurons during neuroinflammation, showing up-regulated miRNAs, their down-regulated target mRNAs, and the confidence of target prediction (Fig. 1C). Gene ontology overrepresentation analyses of target mRNAs revealed biological process terms associated with synapse organization, structure and activity, dendrite development and axonogenesis, neurotransmitter and vesicle transport, as well as membrane potential regulation and ion transmembrane transport. Kyoto Encyclopedia of Genes and Genomes (KEGG) pathway terms revealed a major contribution to hormone signaling such as insulin or growth hormones as well as to calcium-dependent pathways (Fig. 1D). Together, we generated a comprehensive miRNA–mRNA network of inflamed motor neurons that revealed a dominant role of miRNAs in tuning synaptic activity and neuronal communication during neuroinflammation.

### Neuroprotective miR-92a is induced by neuroinflammation and excitotoxicity

**Fig. 2.**
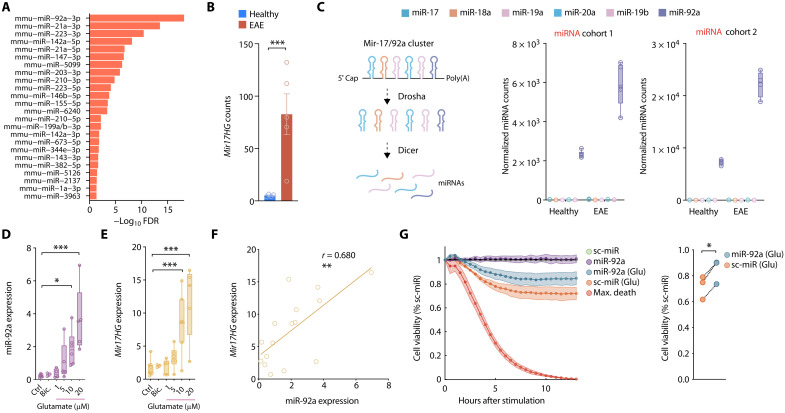
Neuroprotective miR-92a is induced by neuroinflammation and excitotoxicity. (**A**) Motor neuronal miRNA candidates identified across the two independent EAE cohorts (miRNA cohort 1: *n* = 5 per group; miRNA cohort 2: *n* = 4 per group) ranked by statistical significance. (**B**) Normalized *Mir17HG* seq-counts of spinal cord motor neurons after GFP-RPL10A IP (TRAP) from healthy and acute EAE (day 15 after immunization) *Chat-EGFP/Rpl10a* mice. (**C**) Scheme of miR-17/92a cluster (left) and normalized sequencing counts of cluster members (right) in motor neurons from healthy and acute EAE mice. (**D**) qRT-PCR of miR-92a (relative to sno234) in primary neurons 24 hours after stimulation with increasing concentrations of glutamate (Glu) or bicuculline (Bic.) (Ctrl, *n* = 8; Bic., *n* = 3; 1 μM Glu, *n* = 5; 5 μM Glu, *n* = 6; 10 μM Glu, *n* = 7; 20 μM Glu, *n* = 5). ROUT outlier analysis; one-way ANOVA, *F*_5,24_ = 7.870, *P* = 0.0002; Dunnett’s test. (**E**) qRT-PCR of *Mir17HG* (relative to *Tbp*) in primary neurons stimulated as in (D). One-way ANOVA, *F*_5,28_ = 10.59, *P* < 0.0001. (**F**) Pearson correlation of relative miR-92a and *Mir17HG* expression assessed by qRT-PCR 24 hours after exposure to different concentrations of glutamate (16 XY pairs, *P* = 0.0019). (**G**) Real-time viability assay 2 days after transfection of primary neurons with 25 nM miR-92a mimic or scrambled miRNA (sc-miR) control. Neurons were either stimulated with glutamate or vehicle control. Each well was normalized to its own baseline luminescence after 5 hours, to the unstimulated sc-miR control (100% cell viability), and to the max death control (5 mM glutamate, 0% cell viability); *n* = 3; paired *t* test, *P* = 0.011.

miR-92a was the most significantly regulated neuronal miRNA during neuroinflammation, which we independently identified in two EAE cohorts (Fig. 2A). Accordingly, we also found its host gene (*Mir17HG*) significantly induced in our mRNA dataset (Fig. 2B). Although miR-92a is a member of the polycistronic miR-17/92 cluster (which consists of six miRNAs; Fig. 2C), we only found miR-92a to be expressed in relevant quantities from that cluster in motor neurons. Notably, miR-92a was also constitutively expressed by motor neurons in the absence of neuroinflammation, indicating potential homeostatic functions of miR-92a (Fig. 2C). To identify the stimuli that result in the induction of neuronal miR-92a during neuroinflammation, we stimulated primary neurons with cytokines that are abundant in EAE and MS tissue (*19*, *20*) or excitotoxic glutamate levels that contribute to neuronal impairment during neuroinflammation (fig. S2A) (*21*). While the cytokines tumor necrosis factor–α (TNF-α), interferon-γ (IFN-γ), and interleukin-1β (IL-1β) had no influence on miR-92a or *Mir17HG* expression (fig. S2, B and C), glutamate exposure led to a dose-dependent increase in both miR-92a and *Mir17HG* (Fig. 2, D and E). A strong positive correlation between *Mir17HG* and miR-92a induction upon glutamate application indicated that transcriptional induction of miR-92a accounted for the increase in miR-92a abundance (Fig. 2F). Accordingly, no significant correlation was identified between *Mir17HG* and miR-92a upon stimulation with proinflammatory cytokines (fig. S2D). Notably, we did not observe any expression changes of miR-92a or *Mir17HG* by bicuculline [γ-aminobutyric acid type A (GABA_A_) receptor antagonist]–mediated (*22*) synaptic activation of glutamate receptors (Fig. 2, D and E).

To assess the functional consequence of neuronal miR-92a induction, we transfected primary neurons with a synthetically stabilized miR-92a oligonucleotide (miRNA mimic) or a scrambled control miRNA. FAM-labeled miRNA mimics were taken up by neurons, as detected by colocalization with MAP2 immunoreactive cells (fig. S2E). Moreover, transfection of primary neurons with miR-92a mimics resulted in a substantial increase of miR-92a as detected by qRT-PCR (fig. S2F). Next, we explored the effects of miR-92a transfection on cell viability of primary neurons by challenging them with proinflammatory cytokines or glutamate. As the treatment with TNF-α, IFN-γ, and IL-1β did not induce sufficient neuronal cell death (fig. S2G), we focused on the effects of glutamate treatment. In the presence of glutamate, we detected that miR-92a–transfected neurons showed significantly improved survival in comparison to control transfected neurons, whereas survival of unchallenged neurons was unchanged (Fig. 2G). Together, we concluded that miR-92a is induced by extrasynaptic glutamate receptor activation and counteracts glutamate-induced neuronal damage.

### *Cpeb3* is repressed by miR-92a and decreased in neuroinflammation

**Fig. 3.**
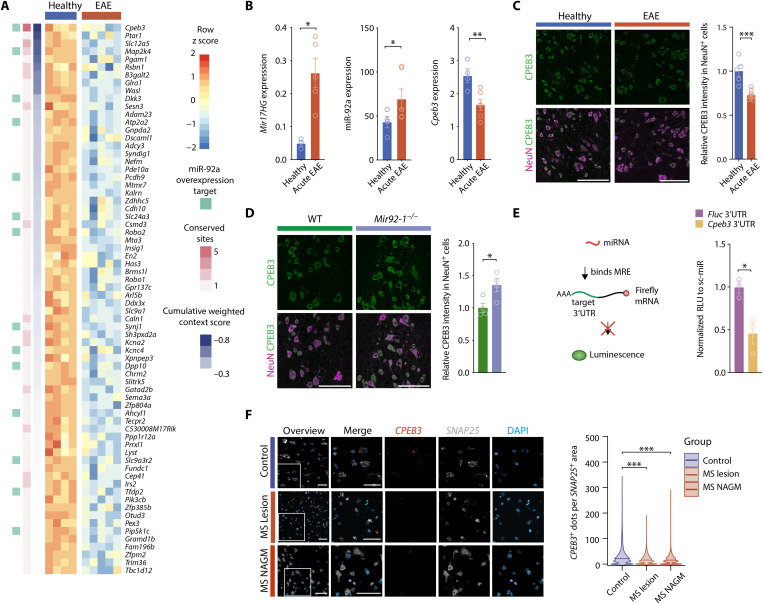
*Cpeb3* is repressed by miR-92a and decreased in neuroinflammation. (**A**) Expression heatmap of predicted miR-92a target mRNAs (CWCS < −0.2; TargetScanMouse 8.0) with significant down-regulation on mRNA level in motor neurons during acute EAE (day 15 after immunization). Annotation for experimentally validated miR-92a targets (*23*) (light green), number of conserved sites (pink), and target prediction confidence (CWCS; blue). (**B**) qRT-PCRs of healthy (*n* = 4) and acute EAE (day 13 after immunization, *n* = 6) cervical spinal cords. ROUT outlier identification, one-tailed Mann-Whitney *U* test. *Mir17HG* (relative to *Tbp)*, *P* = 0.0179; miR-92a (relative to miR-384), *P* = 0.0333; *Cpeb3* (relative to *Tbp)*, *P* = 0.0095. (**C**) Relative mean fluorescence intensity (MFI) of CPEB3 stainings in NeuN-positive cells of healthy (*n* = 5) and acute EAE (day 15 after immunization, *n* = 8) spinal cords. One-tailed Mann-Whitney *U* test, *P* = 0.0008. Scale bars, 100 μm. (**D**) Relative CPEB3 MFI in NeuN-positive cells of *Mir92-1^−/−^* and wild-type littermate control spinal cords. Student’s *t* test, *P* = 0.027, *n* = 4. Scale bars, 100 μm. (**E**) Reporter assay in N2a cells cotransfected with *Cpeb3* 3′UTR or control 3′UTR-containing *firefly* luciferase (FluC) and *renilla* luciferase (RluC) and miR-92a mimic or sc-miR control. FluC is normalized to RluC (RLU) and to the respective sc-miR control. Student’s *t* test, *P* = 0.0168, *n* = 3. (**F**) RNAScope of brain sections from cortical MS lesions, MS NAGM, and non-MS control cortex (control). Scale bars, 50 μm. *CPEB3*-positive dots per *SNAP25*-positive cell area (control *n* = 85,324 neurons; MS NAGM *n* = 88,286 neurons; MS lesion *n* = 96,760 neurons). Mann-Whitney *U* test against control, both *P* < 0.0001.

Having established a neuroprotective role of miR-92a, we more systematically explored predicted miR-92a targets as potential mediators of neuroprotection. We identified 70 predicted miR-92a targets and ranked them by target prediction confidence (Fig. 3A). We further annotated experimentally validated miR-92a targets (*23*), the number of conserved miRNA-binding sites, and the target prediction confidence (Fig. 3A). Next, we performed gene ontology overrepresentation analyses of the 70 target mRNAs and uncovered terms related to regulation of neuronal morphology, hormone, and ion homeostasis, particularly in regard to potassium and calcium signaling (fig. S3A). Among the predicted targets, *Cpeb3* showed that the highest prediction confidence (CWCS = −0.82) harbored the highest number of conserved miRNA-binding sites and was consistent with a miR-92a overexpression study (Fig. 3A) (*23*). Using another independent cohort of wild-type EAE mice (day 13 after immunization), we confirmed that *Mir17HG* and miR-92a induction during neuroinflammation was associated with a significant down-regulation of *Cpeb3* (Fig. 3B). In the same cohort, we also confirmed a significant suppression of CPEB3 on protein level in inflamed spinal cords by immunoblotting (fig. S3B). Further, spinal cords of EAE mice with acute disease (day 15 after immunization) showed neuron-specific suppression of CPEB3 protein within NeuN-immunoreactive cells in comparison to healthy wild-type mice (Fig. 3C). To establish *Cpeb3* as a neuronal miR-92a target in vivo, we investigated the expression of CPEB3 in NeuN-immunoreactive cells of mice deficient for miR-92a in comparison to wild-type littermates. Of note, we observed that mice lacking neuronal miR-92a expression showed an increase in CPEB3 protein levels, supporting that miR-92a negatively regulates CPEB3 expression in neurons (Fig. 3D). To assess silencing of *Cpeb3* by direct binding of miR-92a to the 3′UTR of *Cpeb3*, we cotransfected Neuro-2a (N2a) cells with a 3′UTR-containing luciferase reporter and synthetic miR-92a mimic or scrambled miRNA control. Relative luciferase luminescence (RLU) was significantly reduced in cells cotransfected with *Cpeb3* 3′UTR-luciferase and miR-92a mimics. This was observed after transfection neither with the control vector nor with scrambled miRNA control–transfected cells (Fig. 3E). To assess the translatability of our findings to human MS, we investigated *CPEB3* transcript abundance in brains of MS patients in comparison to control individuals by using RNAScope in situ hybridization. We found that, similar to murine EAE, *CPEB3* levels were significantly decreased in neurons, as denoted by *SNAP25* positivity, of chronic MS lesions as well as of MS normal-appearing gray matter (NAGM) (Fig. 3F).

Together, we identified *Cpeb3* as a key target of miR-92a in inflamed neurons and observed a robust reduction of *CPEB3* in EAE mice and MS patients. Our findings suggest that neuroinflammation leads to an induction of miR-92a and consecutive AGO2-dependent posttranscriptional repression of *Cpeb3* (fig. S3D).

### *Cpeb3* deletion promotes neuronal resistance to excitotoxicity

**Fig. 4.**
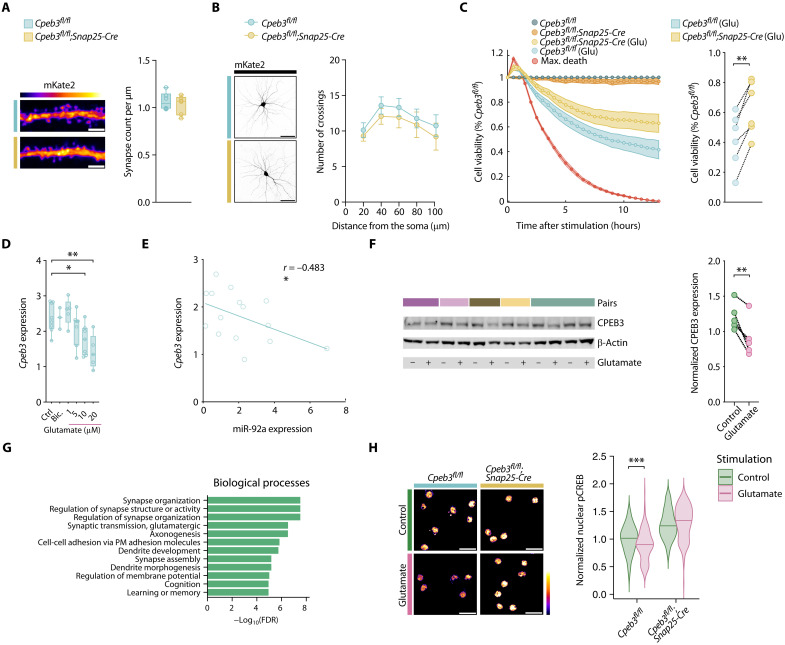
*Cpeb3* deletion promotes neuronal resistance to excitotoxicity. (**A**) Synapse density of mKate2-transfected neurons from *Cpeb3^fl/f^*;*Snap25-Cre* and *Cpeb3^fl/fl^* mice (*n* = 6). Student’s *t* test, *P* = 0.5404. Scale bar, 3 μm. (**B**) Sholl analysis of mKate2-transfected neurons from *Cpeb3^fl/f^*;*Snap25-Cre* and *Cpeb3^fl/fl^* mice (*n* = 6). Two-way ANOVA; Šídák’s test. Scale bar, 50 μm. (**C**) Real-time viability assay of neurons derived from *Cpeb3^fl/fl^*;*Snap25-Cre* and *Cpeb3^fl/fl^* mice stimulated with either glutamate or vehicle control. Each well is normalized to its own baseline luminescence after 5 hours, to the unstimulated *Cpeb3^fl/fl^* control (100% cell viability), and to the max death control (5 mM glutamate, 0% cell viability); *n* = 6; paired *t* test, *P* = 0.0010. (**D**) qRT-PCR of *Cpeb3* (relative to *Tbp*) after 24 hours of stimulation with indicated concentrations of glutamate or bicuculline (25 μM). One-way ANOVA, *F*_5,29_ = 5.384, *P* = 0.0013; Dunnett’s test, *P* < 0.05. (**E**) Pearson correlation of relative miR-92a and *Cpeb3* expression measured by qRT-PCR after exposure to different glutamate concentrations (number of XY pairs, 15, *P* = 0.0340). (**F**) Immunoblotting against CPEB3 (relative to β-actin) of neurons after glutamate treatment (10 to 20 μM). Student’s *t* test, *P* = 0.0095; *n* = 5. (**G**) Gene ontology gene set enrichment analysis of neurons derived from *Cpeb3^fl/fl^*;*Snap25-Cre* versus *Cpeb3^fl/fl^* control mice (*n* = 4) with resulting terms ordered by statistical significance (FDR). (**H**) Nuclear pCREB staining intensity of unstimulated and glutamate-treated (5 μM for 24 hours) neurons (*Cpeb3^fl/fl^* control *n* = 136 nuclei, glutamate *n* = 173 nuclei from *n* = 4 mice; *Cpeb3^fl/f^*;*Snap25-Cre* control *n* = 216 nuclei, glutamate *n* = 180 nuclei from *n* = 5 mice). Scale bar, 25 μm. Mean values normalized to *Cpeb3^fl/f^* unstimulated control. Mann-Whitney *U* test against control, (*Cpeb3^fl/fl^*) *P* < 0.0004, (*Cpeb3^fl/f^*;*Snap25-Cre*) *P* < 0.0970.

To assess the effect of *Cpeb3* repression by miR-92a, we explored the impact of neuron-specific *Cpeb3* deletion. We generated neuron-specific *Cpeb3* knockout mice by crossing floxed *Cpeb3* mice (*24*) with the neuronal Cre driver line *Snap25-IRES2-Cre* (*25*). We tested *Cpeb3* expression in primary neurons derived from *Cpeb3*-deficient mice (*Cpeb3^fl/fl^;Snap25-Cre*) and detected significantly reduced transcript and protein levels in comparison to *Cpeb3*-proficient mice (*Cpeb3^fl/fl^*) (fig. S4, A and B). While CPEB3 has been described as modulator of synaptic plasticity (*24*, *26*), we did not observe developmental alterations in *Cpeb3*-deficient neurons, as tested by synaptic density (Fig. 4A) and dendritic arborization (Fig. 4B) of neuronal cultures. Notably, by exposing neurons to glutamate, we detected significantly increased survival of *Cpeb3*-deficient neurons in comparison to *Cpeb3*-proficient neurons (Fig. 4C). Unchallenged neurons did not show any differences in survival. Consistent with our observation that miR-92a and *Mir17HG* showed a dose-dependent increase in expression after glutamate exposure (Fig. 2, D to F), *Cpeb3* transcript expression showed a dose-dependent decrease after glutamate exposure that was negatively correlated with miR-92a expression and was unaffected by bicuculline treatment (Fig. 4, D and E). We also observed a significant decrease of CPEB3 protein after glutamate exposure (Fig. 4F). RNA sequencing and gene ontology overrepresentation analysis of primary *Cpeb3*-deficient neurons in comparison to wild-type neurons revealed biological processes associated with synapse organization, synapse activity, and glutamatergic synaptic transmission (Fig. 4G and fig. S4C) and KEGG pathway terms related to neurodegenerative diseases and oxidative phosphorylation (fig. S4D), partly reflecting the biological themes derived from our miRNA–mRNA network. Supporting a role for CPEB3 in regulating glutamate responses, reduction of the phosphorylation of cyclic adenosine monophosphate–responsive element binding protein (pCREB), an indicator for glutamate receptor–mediated excitotoxicity (*22*), was alleviated in primary neurons that were deficient for *Cpeb3* (Fig. 4H). Together, we show that *Cpeb3* transcript and protein abundance in neurons are reduced by glutamate excitotoxicity resulting in maintained pCREB levels and improved survival.

### *Cpeb3* deletion enhances neuroaxonal resistance and ameliorates clinical disability in EAE

**Fig. 5.**
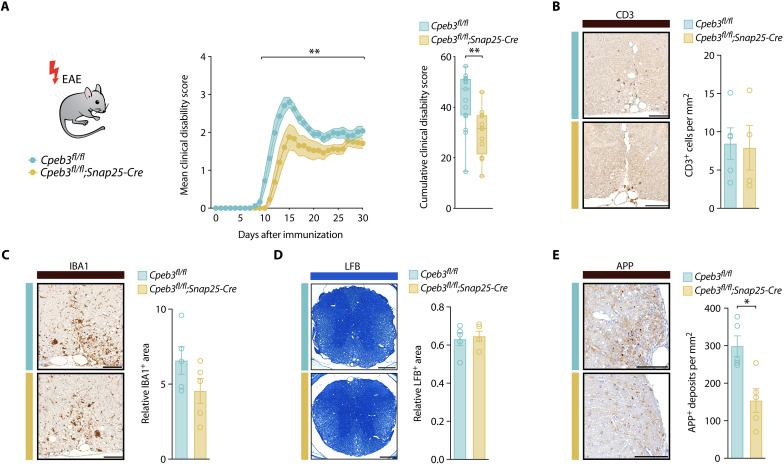
*Cpeb3* deletion enhances neuroaxonal resistance and ameliorates clinical disability in EAE. (**A**) Disease course of EAE *Cpeb3^fl/fl^*;*Snap25-Cre* mice *(n* = 12) and *Cpeb3^fl/fl^* littermate controls (*n* = 17) showing mean clinical disability of pooled data from two independent experiments. Mann-Whitney *U* test of the area under the curve (AUC) of each animal, *P* = 0.0018. (**B**) Immunohistochemical staining and quantification of CD3^+^ cells in cervical spinal cords of *Cpeb3^fl/fl^*;*Snap25-Cre* versus *Cpeb3^fl/fl^* control mice at EAE day 30 after immunization. CD3^+^ cells are normalized to the spinal cord area. Mann-Whitney *U* test, *P* > 0.9999, *n* = 5. Scale bar, 100 μm. (**C**) Immunohistochemical staining and quantification of the IBA^+^ area in cervical spinal cord of *Cpeb3^fl/fl^*;*Snap25-Cre* versus *Cpeb3^fl/fl^* control mice at EAE day 30 after immunization. Mann-Whitney *U* test, *P* > 0.3905, *n* = 5. Scale bar, 100 μm. (**D**) Staining of myelin with Luxol-Fast-Blue (LFB) in cervical spinal cords of *Cpeb3^fl/fl^*;*Snap25-Cre* versus *Cpeb3^fl/fl^* control mice at EAE day 30 after immunization. The LFB^+^ area is normalized to the spinal cord area. Student’s *t* test, *P* = 0.8413, *n* = 5. Scale bar, 250 μm. (**E**) Immunohistochemical staining and quantification of injured APP^+^ axons in cervical spinal cord white matter of *Cpeb3^fl/fl^*;*Snap25-Cre* versus *Cpeb3^fl/fl^* control mice at EAE day 30 after immunization. Mann-Whitney *U* test, *P* = 0.0159, *n* = 5. Scale bar, 100 μm.

Next, we assessed the effect of neuronal *Cpeb3* deletion during neuroinflammation in vivo. After validating neuron-specific deletion of CPEB3 in *Cpeb3^fl/fl^;Snap25-Cre* knockout mice (fig. S5, A and B), we confirmed the absence of neurodevelopmental deficits by analyzing neuronal numbers and myelination in the spinal cord (fig. S5, C and D). We then immunized animals for EAE induction and observed a significant reduction of the cumulative clinical disability in mice with a neuron-specific deletion of *Cpeb3* in comparison to *Cpeb3^fl/fl^* littermate controls (Fig. 5A). Whereas T cell infiltration and microglia activation as well as myelination were not different in the cervical spinal cord of *Cpeb3^fl/fl^;Snap25-Cre* and *Cpeb3^fl/fl^* littermate controls at chronic EAE (day 30 after immunization) (Fig. 5, B to D), the number of amyloid-β precursor protein (APP)–positive axons, as a measure of injured axons, was significantly decreased in mice with neuron-specific *Cpeb3* deletion (Fig. 5E).

Together, we show that neuron-specific *Cpeb3* deletion reduces neuroaxonal damage during CNS inflammation, resulting in ameliorated clinical disability of EAE mice without affecting immune cell infiltration and activation. Our results indicate that the stress-induced neuronal induction of miR-92a and consecutive repression of CPEB3 constitutes a neuron-intrinsic axis to reinforce neuronal resistance toward excitotoxicity and inflammatory stress.

## DISCUSSION

Neuroinflammation is driving neurodegeneration in MS and other neurological pathologies (*27*). However, despite the urgent unmet clinical need for neuroprotective treatments in neuroinflammatory conditions, a detailed understanding of how neurons withstand inflammation-induced impairment is lacking. Here, we leveraged miRNA–mRNA network profiling of inflamed neurons to assess the role of miRNAs in shaping neuronal transcriptional responses during neuroinflammation. First, we identified neuronal miRNAs with robust regulation across two independent cohorts of mice undergoing EAE. By integrating the predicted targets of these 24 miRNAs with experimentally validated down-regulated mRNAs, derived from neuron-specific mRNA sequencing, we constructed a comprehensive miRNA–mRNA network of neuroinflammation comprising 681 interactions. Within this network, we identified the neuroprotective miR-92a–CPEB3 axis that shields neurons against glutamate-induced excitotoxicity and improved the clinical outcome in the EAE animal model.

MiRNAs work as higher-level transcriptional regulators with the capacity to maintain and restore cellular homeostasis by specific silencing of target mRNA transcripts across cellular compartments (*10*). By controlling gene expression networks (*14*), miRNAs can increase cellular resistance against internal and external perturbations (*10*). Despite their accepted role as gatekeepers of cellular homeostasis, little is known about their function in neurons that are challenged with the detrimental cues present in inflammatory microenvironments. It is conceivable that the miRNA–mRNA network of neurons might adapt in an attempt to preserve neuronal homeostasis, thereby exposing neuroprotective pathways that can be further exploited for neuron-directed therapy. Following this rationale, we generated a comprehensive dataset that allowed the construction of a miRNA–mRNA network, which we consider a rich resource to explore neuronal defense mechanisms to maintain homeostasis in the face of an inflammatory challenge.

From this network, we extracted miR-92a as the most prominent miRNA candidate that showed robust induction across independent experimental cohorts. A similar approach using laser microdissection of EAE motor neurons and retinal neurons reported 14 differentially regulated miRNAs implicated in neuronal repair and regeneration (*28*). However, this study did not find changes in miR-92a, possibly due to methodological differences of whole-cell laser microdissection (*29*) versus profiling of AGO2-bound miRNA (*16*). While a functional role of miR-92a has been proposed in neurogenesis, anxiety, and depression-like behaviors (*30*), we here provide evidence for a neuroprotective response of miR-92a to inflammatory stress.

We found that expression of *Mir17HG* and levels of miR-92a were significantly induced upon glutamate challenge in neuronal cultures. Given that neuroinflammation triggers the release of glutamate from neurons, infiltrating immune cells and microglia (*21*, *31*), the rise in extracellular glutamate might present a central trigger for the induction of miR-92a also in inflamed neurons in vivo, especially since other inflammatory mediators including TNF-α, IFN-γ, or IL-1β failed to induce miR-92a expression. Glutamate excitotoxicity is a prominent cause for neuronal injury during neuroinflammation in MS and EAE. Increased levels of extracellular glutamate have been identified in the cerebrospinal fluid and brains of MS patients and have been associated with neurodegeneration (*32*, *33*). Elevated glutamate has also been identified in brains of EAE mice, and modulation of glutamate receptor activity has been proven effective in ameliorating neuroaxonal damage in EAE (*21*, *34*). Notably, bicuculline treatment, which blocks GABA_A_ receptors and thereby evokes an increase of synaptic glutamate release (*22*), also failed to induce miR-92a, suggesting that the activation of synaptic glutamate receptors might not be sufficient to drive miR-92a expression. Instead, receptors that sense extracellular glutamate excess and have been shown to drive neuronal excitotoxicity (*22*, *35*), i.e., by engaging extrasynaptic glutamate receptors, are suitable candidates to trigger neuronal miR-92a expression.

Further, we demonstrate that the application of miR-92a mimics protects primary neurons against glutamate-induced cell death. While a functional role of miR-92a has not been described in MS, miR-92a has been identified to be increased in the serum of MS patients and has been correlated with the extent of neurodegeneration detected by magnetic resonance imaging (*36*). However, the origin and functional role of increased miR-92a remained speculative. Notably, adeno-associated virus (AAV)–mediated overexpression of the miR-17/92 cluster has been shown to rescue motor neuronal survival in a mouse model of amyotrophic lateral sclerosis and to protect neurons expressing mutated human superoxide dismutase (*SOD*)^+/L144F^ from degeneration. Accordingly, deletion of the miR-17/92 cluster induced apoptosis and led to motor neuronal degeneration (*13*). While in this study the specific contribution of individual miRNAs from the miR-17/92 cluster remained unclear, our data clearly identified miR-92a as a neuroprotective entity, which has also been demonstrated by treating mice with synthetic miR-92a oligonucleotides after spinal cord injury (*37*). Moreover, the miR-17/92 cluster has also been shown to be active in CNS cell types other than neurons. A positive influence on oligodendrocyte numbers (*38*) has been demonstrated, which could indirectly affect neuronal function during neuroinflammation. Together, these data support the neuroprotective potential of miR-92a to prevent or ameliorate neurodegeneration in different neurological pathologies.

To elucidate neuroprotective downstream effects of miR-92a, we capitalized on our miRNA–mRNA network and extracted *Cpeb3* as the most prominent miR-92a target in inflamed neurons. In support of a posttranscriptional silencing of *Cpeb3* by miR-92a (*39*), we observed an induction of neuronal CPEB3 protein expression in miR-92a knockout spinal cords and found a direct interaction of miR-92a with *Cpeb3* 3′UTR. In line with a neuroprotective effect of *Cpeb3* silencing, we found reduced glutamate-induced cell death and ameliorated clinical disability in *Cpeb3* knockout neuronal cultures and neuron-specific *Cpeb3* knockout animals undergoing EAE, respectively. Notably, we detected a nuclear pCREB shutoff in wild-type neurons upon glutamate treatment, which was absent in *Cpeb3*-deficient neurons. As CREB is an important transcription factor mediating neuronal survival and plasticity, and shutoff is associated with glutamate-induced synaptic dysfunction and neuronal cell death (*22*), these results give a mechanistic explanation of the increased survival of *Cpeb3*-deficient neurons upon excitotoxic glutamate challenge. While it has been demonstrated that miR-92a controls CPEB3 levels in contextual fear memory formation in the hippocampus of mice (*40*), CPEBs including CPEB3 have not yet been associated with neuroinflammation or MS pathology. Supporting the translational relevance for MS, we uncovered that neuronal *CPEB3* mRNA was reduced in NAGM and chronic lesions in brains of MS patients in comparison to healthy control individuals, suggesting that CPEB3 repression in response to neuroinflammation is conserved across species. Supporting the potential of targeting CPEB3, a decrease in CPEB3 levels has been shown to boost neurotrophic signaling (*41*), to ameliorate oxidative stress (*42*), and to increase proteasome activation (*43*). Moreover, CPEB3 contains a prion-like domain that has been shown to form amyloid-like aggregates upon neuronal activation (*44*) that are toxic in vitro (*45*). Together, these findings advocate the further development of CPEB3-directed therapies with potential broad implication for neuroinflammation and other neurological diseases that benefit from supporting neuronal resistance.

Together, we harnessed cell type–specific profiling to construct a neuronal miRNA–mRNA network of neuroinflammation, which allowed us to identify a neuroprotective miR-92a–CPEB3 axis. We found that this axis mitigates neuronal cell death upon glutamate excitotoxicity in primary neurons and improves the clinical outcome in a mouse model of MS. Further, we show the translational potential of our findings, as neuronal regulation of *CPEB3* mRNA in inflamed neurons was consistent across murine EAE and human MS. Thus, therapeutic targeting of CPEB3 transpires as a promising approach to support neuronal survival during neuroinflammation in MS and potentially other neuroinflammatory conditions.

## MATERIALS AND METHODS

### Animals

All mice, *C57BL/6J wild-type* (The Jackson Laboratory), *R26-LSL-tAgo2* (*16*), *Chat-IRES-Cre* (strain no. 006410, The Jackson Laboratory), *R26-LSL-tAgo2;Chat-IRES-Cre*, *Chat-EGFP/Rpl10a* (*17*), *Mir92-1^−/−^* (*46*) (strain no 027320, The Jackson Laboratory), *Snap25-IRES2-Cre-D* (strain no. 023525, The Jackson Laboratory), *Cpeb3^fl/fl^* (*24*) (provided by Y.-S.H., Taiwan, and transferred from A. Susor, Czech Republic), and *Cpeb3^fl/fl^*;*Snap25-Cre* were housed and bred at the Central Animal Facility at the University Medical Center Hamburg-Eppendorf (UKE). Mice were provided with food and water ad libitum. One to 2 weeks before starting an experiment, the mice were transferred into the experimental barrier and kept in individually ventilated cages under specific pathogen–free conditions. We used adult female and male mice for biochemical analyses. Because of their more consistent disease course, we used female mice in EAE experiments. All efforts were made to minimize animal numbers and their suffering.

### Experimental autoimmune encephalomyelitis

Mice were immunized subcutaneously with 200 μg of MOG_35–55_ (Peptides & Elephants) in complete Freund’s adjuvant (BD Difco Adjuvants, catalog no. DF0639-60-6) containing *Mycobacterium tuberculosis* (4 mg ml^−1^; BD Difco Adjuvants, catalog no. DF3114-33-8). The mice were injected intraperitoneally with 200 ng of pertussis toxin (Sigma-Aldrich, catalog no. 516560s) on the day of immunization and 48 hours later. Mice were provided with softened food pellets and DietGel Recovery gel (Ssniff, catalog no. H007-72065). Clinical disability of mice was assessed daily by a blinded experimenter (0, no clinical symptoms; 1, paresis of the tail; 2, paresis of the hindlimbs; 3, partial paralysis of the hindlimbs; 3.5, paralysis of the hindlimbs; 4, paralysis of the hindlimbs and paresis of the front limbs; 5, premorbidity or death). Mice reaching a clinical score of ≥4 were euthanized according to the regulations of the local Animal Welfare Act. All EAE experiments were carried out with littermate controls. The EAE scores were presented as mean values ± the SEM per day. Animals with no disease symptoms were excluded from the analysis. Missing values were conservatively imputed as the mean clinical score of the respective experimental group on that day. EAE samples were obtained on day 12 (miRNA sequencing) or day 15 after immunization (mRNA sequencing) from animals with a clinical score of ≥2.5. Littermates without immunization were used as healthy control animals.

### Translating ribosome affinity purification

Translating ribosome affinity purification (TRAP) (*47*) was carried out as described previously (*8*). Briefly, female *Chat-EGFP/Rpl10a* (*17*) mice were anesthetized with an overdose (15 μl/g body weight) of ketamine/xylazine (12/1.6 mg ml^−1^, intraperitoneally) solution in phosphate-buffered saline (PBS) and perfused with dissection buffer containing 1× Hanks’ balanced salt solution, 2.5 mM Hepes-KOH (pH 7.4), 35 mM glucose, and 4 mM NaHCO_3_. Cervical spinal cords of three female *Chat-EGFP/Rpl10a* mice were pooled per sample and homogenized with a glass potter (Satorius) in lysis buffer containing 20 mM Hepes-KOH (pH 7.4), 150 mM KCl, 5 mM MgCl_2_, 0.5 mM dithiothreitol (DTT), cycloheximide (CHX; 100 μg ml^−1^), RNasin (40 U ml^−1^, Promega), and SUPERase-In ribonuclease (RNase) inhibitor (20 U ml^−1^, Invitrogen) and centrifuged for 10 min at 2000*g* at 4°C. The supernatant was incubated in a final concentration of 1% NP-40 and 30 mM 1,2-diheptanoyl-*sn*-glycero-3-phosphocholine (Avanti Polar Lipids) for 5 min on ice and centrifuged for 10 min at 20,000*g* at 4°C. The supernatant was further processed to either isolate mRNAs directly (input) or purify GFP-RPL10A–associated mRNAs by IP. GFP-IP was performed with monoclonal rabbit anti-GFP antibodies (Htz-GFP19C8 and Htz-GFP19F7, Memorial Sloan Kettering Cancer Center Monoclonal Antibody Core Facility) that were precoupled to Dynabeads MyOne Streptavidin T1 (Invitrogen) by using Pierce recombinant biotinylated Protein L (Thermo Fisher Scientific) at 4°C overnight. Before anti-GFP coupling, beads were blocked in 3% immunoglobulin G– and protease-free bovine serum albumin (BSA; Jackson ImmunoResearch) for 35 min at room temperature. Freshly coated beads were washed in low-salt buffer containing 20 mM Hepes, 150 mM KCl, 5 mM MgCl_2_, 1% NP-40, 0.5 mM DTT, and CHX (100 μg ml^−1^) . After incubation with sample supernatant, beads were washed with high-salt buffer [20 mM Hepes, 350 mM KCl, 5 mM MgCl_2_, 1% NP-40, 0.5 mM DTT, and CHX (100 μg ml^−1^)]. IPs and inputs were subjected to phenol/guanidine-based RNA extraction (TRIzol, Invitrogen). RNA was precipitated in 0.3 M sodium acetate, an equal volume of 2-propanol, and GlycoBlue Coprecipitant (50 ng μl^−1^, Invitrogen) at −80°C overnight. After centrifugation at 13,000*g* for 15 min, pellets were washed twice with ice-cold EtOH (70%), air-dried, and resuspended in RNase-free water. RNA was further purified using RNeasy MinElute Spin columns (RNeasy Micro Kit, Qiagen) with on-column deoxyribonuclease (DNase) I (Qiagen) digestion according to the manufacturer’s protocol. For higher RNA yields, all steps were carried out in nonstick Ambion RNase Microfuge Tubes (Invitrogen). The RNA was eluted in 14 μl of RNase-free water and stored at −80°C. Every step was carried out on RNaseZap (Ambion) cleaned laboratory workbenches and tools as well as RNase- and DNase-free consumables.

### miRNA tagging and affinity purification

The miRNA tagging and affinity purification protocol (*16*) was adjusted to achieve the highest comparability with TRAP. Cervical spinal cords of three female *R26-LSL-tAgo2;Chat-IRES-Cre* mice were pooled per sample for GFP-AGO2 IP, which was performed as described above for TRAP with minor modifications. After washing of beads, IPs and inputs were subjected to phenol/guanidine-based RNA extraction (QIAzol, Qiagen) according to the manufacturer’s protocol. After a centrifugation at 12,000*g* for 15 min at 4°C, the upper colorless phase was carefully removed and mixed with 1.5 volumes of ~100% EtOH. Purification of miRNAs was achieved by RNeasy MinElute spin columns (miRNeasy Micro Kit, Qiagen) according to the manufacturer’s protocol. miRNA was eluted in 14 μl of RNase-free water and stored at −80°C.

### Small RNA and mRNA sequencing and computational analysis

Small RNA and mRNA sequencing was performed by the NGS Integrative Genomics Core Unit (NIG) in Göttingen. Small RNA sequencing libraries were prepared using the TruSeq Small RNA Library Prep Kit (Illumina). mRNA libraries were prepared using the NEBNext Ultra RNA Library Prep Kit for Illumina (New England Biolabs) with minor modifications. Libraries were sequenced on Illumina HiSeq 2000 and 4000 machines generating 50–base pair single-end reads. mRNA sequencing of cortical neuronal cultures of *Cpeb3^fl/fl^;Snap25-Cre* animals and *Cpeb3^fl/fl^* controls was performed by Novogene. Libraries were prepared using the Novogene NGS RNA Library Prep Set (Novogene) and sequenced on an Illumina NovaSeq 6000 machine generating 150–base pair paired-end reads.

#### Small RNA sequencing analysis

The raw sequencing reads were adaptor trimmed, aligned, and counted with OASIS 2.0 (https://oasis.ims.bio/) (*48*). miRNA counts were analyzed by DESeq2 (v1.36.0) (*49*) calling miRNAs with a false discovery rate (FDR)–adjusted *P* value of <0.05 and a mean expression change of >50% differentially expressed. For the identification of candidate miRNAs, two independent cohorts were analyzed separately (cohort 1: five healthy versus five EAE day 12; cohort 2: four healthy versus four EAE day 12). Twenty-four miRNA candidates were independently identified in both cohorts using the statistic criteria described above (table S1). Candidate miRNAs were ranked on the basis of the maximal minus log_10_ FDR-adjusted *P* value across both cohorts, i.e., miRNAs with the lowest *P* values across cohorts were ranked as top hits (Fig. 2A). Expression heatmaps were generated from expression values after variance stabilizing transformation (DESeq2) using the R package tidyheatmaps (v0.1.0, https://github.com/jbengler/tidyheatmaps). Additional plotting was done using the R package ggplot2 (v3.3.6). Raw data are available under GSE220950.

#### mRNA sequencing analysis

The raw sequencing reads were aligned to the Ensemble mouse reference genome (GRCm38r79; mm10) using STAR (v2.5.2b) with default parameters, and overlap with annotated gene loci were counted with featureCounts (v1.5.1). Samples with less than 1 M assigned reads were excluded. Remaining samples were analyzed with DESeq2 (v1.36.0) (*49*) calling mRNAs with an FDR-adjusted *P* value of <0.05 and a mean expression change of >50% differentially expressed (tables S2 and S3). Raw data are accessible under GSE220951 and GSE224558.

#### miRNA-mRNA network analysis

Predicted targets of the 24 miRNA candidates were extracted from TargetScanMouse 8.0 (32,253 predicted miRNA-mRNA interactions) (*18*). The confidence level of predictions was stratified by using the CWCS. Moderate-confidence (CWCS between −0.2 and −0.4) and high-confidence targets (CWCS lower than −0.4) were retained (5720 predicted interactions) and further filtered to only include mRNAs that were significantly down-regulated in our EAE day 15 TRAP mRNA sequencing dataset (2145 down-regulated mRNAs) using the statistic criteria described above. Thereby, we focused our analysis on predicted targets that showed a reduction in transcript abundance, as would be expected in case of effective miRNA-mediated silencing (*50*). This conservative approach resulted in 681 predicted interactions that were used to construct a miRNA–mRNA network (Fig. 1C) using the R packages network (v1.18.0) and ggnetwork (v0.5.10). The size of target nodes reflects the target confidence (CWCS), and high confidence targets (CWCS lower than −0.4) are additionally labeled with their respective gene name (table S4).

#### Gene ontology analysis

Overrepresentation analysis of unranked gene lists was performed using the functions enrichGO and enrichKEGG from the package clusterProfiler (v4.4.4) to assess enrichment of gene ontology and KEGG pathway terms, respectively. Gene set enrichment analysis of gene expression signatures was performed using the functions gseGO and gseKEGG. The top 12 enriched terms were plotted as bar graphs ranked by statistical significance.

#### miR-92a target analysis

The seed region of miR-92a-3p “AUUGCAC” is shared by a group of miRNAs (miR-25-3p/32-5p/92-3p/363-3p/367-3p) referred to as “miR-25 seed family” (*51*). For miR-92a target analysis, predicted targets of the miR-25 seed family were filtered to only contain moderate- and high-confidence targets (CWCS lower than −0.2) that showed a significant down-regulation in our EAE day 15 TRAP mRNA sequencing dataset (see above). The resulting 70 predicted targets were ranked by CWCS and plotted as heatmap of mRNA expression values after variance stabilizing transformation (DESeq2) using the R package tidyheatmaps (v0.1.0, https://github.com/jbengler/tidyheatmaps). The heatmap was further augmented by including information about experimentally validated miR-92a targets [identified by miR-92a overexpression (*23*)], the CWCS, and the number of conserved sites, as shown in the respective annotation columns (Fig. 3A).

### qRT-PCR for mRNA and miRNA

#### Nucleic acid isolation

Cell pellets or mouse tissue (~20 mg) was lysed in RLT buffer (RNeasy Mini Kit, Qiagen) containing β-mercaptoethanol (1:100) and subsequently homogenized by QIAshredder columns (Qiagen). Mouse tissue was additionally homogenized in a glass douncer. RNA was purified from the column flow-through with silica gel membrane containing spin columns (RNeasy Mini Kit) according to the manufacturer’s protocol. The RNA was eluted in 30 μl of RNase-free H_2_O and stored at −80°C. Every step was carried out on RNaseZap (Ambion) cleaned laboratory workbenches and tools as well as RNase- and DNase-free consumables. The total RNA content was measured by a spectrophotometer (NanoDrop, Thermo Fisher Scientific).

#### Universal miRNA transcription

miRNAs were universally reverse-transcribed by the TaqMan Advanced miRNA cDNA Synthesis Kit (Thermo Fisher Scientific, catalog no. A28007) and detected with specific TaqMan Advanced miRNA assays for quantification by qRT-PCR. For each sample, 10 ng of total RNA was reverse-transcribed and amplified according to the manufacturer’s protocol. The RT and miR-Amp products were stored at −20°C. The qRT-PCR reaction mix was prepared with miRNA-specific TaqMan Advanced miRNA assays (table S5) in RNase-free H_2_O and TaqMan Fast Advanced Master Mix (Thermo Fisher Scientific, catalog no. 4444557) according to the manufacturer’s protocol.

#### miRNA-specific transcription for miR-92a

Since no specific TaqMan Advanced miRNA Assay existed to detect miR-92a, another TaqMan qRT-PCR system was used, which is based on miRNA-specific RT for each miRNA or respective miRNA control. RT was performed with the TaqMan miRNA RT Kit (Thermo Fisher Scientific, catalog no. 4366596). For each reaction, 10 ng of total RNA was reverse-transcribed by MultiScribe Reverse Transcriptase (50 U μl^−1^) with miRNA-specific RT primers according to the manufacturer’s protocol. The RT product was stored at −20°C. The qRT-PCR reaction mix was prepared with miRNA-specific TaqMan miRNA Assays (table S5) in RNase-free H_2_O and TaqMan Universal PCR Master Mix II with uracil-*N*-glycosylase (Thermo Fisher Scientific, catalog no. 4440038) according to the manufacturer’s protocol.

#### mRNA transcription

mRNAs were reverse-transcribed with the High-Capacity RNA-to-cDNA Kit (Thermo Fisher Scientific, catalog no. 4387406). For each sample, 80 ng of total RNA was universally reverse-transcribed by RT Enzyme Mix (MultiScribe) and an RT buffer mix with oligo d(T)16 and random octamer primer in RNase-free H_2_O according to the manufacturer’s protocol. The RT product was stored at −20°C. The qRT-PCR reaction mix was prepared with gene-specific FAM dye–labeled TaqMan MGB probes with two unlabeled PCR primer (TaqMan Gene Expression Assays) (table S5) and TaqMan Gene Expression Master Mix (Thermo Fisher Scientific, catalog no. 4370074) according to the manufacturer’s protocol. qRT-PCR reaction plates (384-well or 96-well plates, Applied Biosystems) were prepared with 8 μl of qRT-PCR reaction mix per well and 2 μl of cDNA.

#### Quantitative RT-PCR

qRT-PCR was performed in triplicates for each sample and each miRNA or mRNA, respectively. qRT-PCR was run on ABI Prism 7900 HT Fast Real-Time PCR System or the Applied Biosystems QuantStudio 6 Flex Real-Time PCR System in comparative *C*_T_ (ΔΔ*C*_T_) run mode according to the manufacturer’s instructions. Analysis was performed with SDS v2.4.1 and RQ Manager v1.2 or QuantStudio v1.3 software. miRNA expression was normalized to the small nucleolar RNA sno234 (*52*) or to miR-384, which was identified as endogenous control for miRNA analyses in EAE, as it showed stable expression among healthy spinal cord, EAE spinal cord, healthy motor neurons, and EAE motor neurons. mRNA expression was normalized to the housekeeping gene *Tbp* (*8*). The quantification of miRNA and mRNA was performed according to 2^–ΔΔ*C*T^ using mean *C*_T_ values from technical triplicates.

### Primary neuronal cultures

Primary cortical mouse neurons were prepared as described previously (*9*). Pregnant *C57BL/6J* or *Cpeb3^fl/fl^;Snap25-Cre* mice were euthanized on gestational day E15.5 to E16.5, and cortices of prenatal mice were isolated and trypsinized in 0.05% EDTA solution (Gibco, catalog no. 25300054) for 6 min at 37°C. Subsequently, trypsinization was stopped by adding sterile-filtered Dulbecco’s modified Eagle’s medium (DMEM)/F-12 (Gibco, catalog no. 21331020) supplemented with 10% fetal bovine serum (FBS) (PAN Biotech, catalog no. P30-3306). Cortices were gently dissociated, and cells were seeded at a density of 1 × 10^5^ cells/cm^2^ on poly-d-lysine (PDL; 50 μg ml^−1^) (Sigma-Aldrich, catalog no. A-003-M) precoated polystyrene multiwell plates (Greiner). Cells were plated in Neurobasal B-27 Plus (Gibco, catalog no. A3653401) and supplemented with penicillin-streptomycin (50 U ml^−1^, Gibco, catalog no. 15070063), 0.05 mM GlutaMAX supplement (Gibco, catalog no. 35050061), and 25 μM glutamate. The next day, cells were treated with 1 μM cytosine arabinoside (Ara-C; Sigma-Aldrich, catalog no. BP383) to inhibit glial cell proliferation. Cells were fed every 4 to 5 days by 50% medium exchange with prewarmed fresh feeding medium (without glutamate). The cortical cells were cultured for 14 to 18 days before further analysis in an incubator at 37°C, 5% CO_2_, and a relative humidity of 98%.

### Neuroinflammatory stress induction

Primary cortical neurons were stimulated with bicuculline (25 μM) (*34*); the proinflammatory cytokines TNF-α, IFN-γ, or IL-1β (100 ng ml^−1^) (*53*); or glutamate bath (1, 5, 10, and 20 μM) for 24 hours (*9*, *34*) to simulate chronic cell stress. Subsequently, neurons were washed twice in ice-cold sterile PBS without Ca/Mg [Dulbecco’s PBS (DPBS)] (PAN Biotech, catalog no. P04-36500) and carefully detached from the cell culture plate surface with cell scrapers (Greiner) in ice-cold DPBS. Neurons were pelleted by centrifugation for 5 min at 4°C. After aspiration of DPBS, neuronal cell pellets were snap-frozen in liquid nitrogen and stored at −80°C.

### Transfection of primary neurons

For analysis of miR-92a function, primary neurons were transfected with 25 nM miRCURY LNA miRNA Mimics (Qiagen, catalog no. 339173) of either miR-92a (YM00470097-ADA) or scrambled control miRNA (YM00479903-ADA) with NeuroMag transfection reagent (Ozbioscience, catalog no. NM50500) according to the manufacturer’s protocol. For analysis of miRNA uptake, 25 nM FAM-labeled miRCURY LNA miRNA Mimics (Qiagen, catalog no. 339173, YM00479903-ADB) were used. Briefly, cells were fed with serum-free medium 24 hours before transfection procedure. The next day, NeuroMag beads were mixed with miRCURY LNA miRNAs in serum-free medium without supplements at room temperature, incubated for 15 min, and carefully dropped onto the cells. The microplate was rocked from side to side for equal distribution and placed on a magnetic plate for 20 min. Half of the medium was replaced with preconditioned medium that has been collected before the transfection procedure. Cells were incubated for 48 hours before further analysis.

For analysis of synaptic density and dendritic arborization, primary neurons at day 5 in vitro were transfected with 0.4 μg of plasmid containing mKate2 fluorophore (provided by T. Oertner, Germany) (*54*) under the control of an hSYN1 promotor in Lipofectamine 2000 reagent according to the manufacturer’s protocol with minor modifications. The reagents were diluted in Neurobasal Plus (Gibco, catalog no. A3653401) without supplements. The DNA-Lipofectamine mixture was carefully replaced by preconditioned neuronal medium after 40 min of incubation. Samples at day 16 in vitro were fixed in 4% paraformaldehyde (PFA) for 15 min, washed in 1 × PBS thrice, and mounted in Mowiol 4-88 (Merck Chemicals). Fluorescent images were acquired by confocal laser scanning microscopy (Fluoview FV1000, Olympus) at ×60 magnification with Z-stacks at 0.3-μm intervals and 512 × 512 resolution and 414 × 414 nm pixel size (Sholl analysis) or 80 × 80 nm pixel size (synaptic density), respectively. Image analysis was performed on the raw data. For Sholl analysis, a maximal projection of all planes was created with Fiji software (*55*), and the radial concentric circles were drawn with the Concentric Circles plugin with a step of 20 μm around the neuronal soma as center. The number of dendritic intersections was counted for each circle. For dendritic spine density, the number of dendritic spines along the stretch of a primary proximal dendrite was divided by the length of the dendritic stretch. The representative images were created in Fiji with linear contrast adjustment (histogram normalization) and median filter.

### Real-time cell viability assay

RealTime-Glo MT cell viability assay (Promega, catalog no. G9711) was performed as described previously (*9*). Briefly, RealTime-Glo reagents were mixed and added to neuronal cultures in 96-well microplate format (μclear, Greiner, catalog no. 655094). Luminescence was acquired with a Spark 10M multimode microplate reader (Tecan) at 37°C and 5% CO_2_ in intervals of 30 min over a total time period of 24 hours. At least five technical replicates per condition were used. After equilibration of luminescence signal (5 hours), glutamate or vehicle was applied (5 to 40 μM). All values were normalized between 0% cell viability (maximum cell death control with 5 mM glutamate) and 100% cell viability (vehicle control) using a customized MATLAB (R2021b) script. For statistics and visualization, the glutamate-treated values of each group were pooled.

### Immunohistochemistry and immunohistopathology

Mice were anesthetized with an overdose (15 μl/g body weight) of ketamine/xylazine (12/1.6 mg ml^−1^, intraperitoneally) solution in PBS followed by 4% PFA perfusion as described previously (*9*). Cervical spinal cords were dissected and postfixated in 4% PFA for 30 min. Then, the spinal cords were either dehydrated and cryoprotected in 30% sucrose solution in PBS for at least 1 to 2 days at 4°C, frozen in embedding solution (Tissue-Tek O.C.T. compound), and cut into 12-μm-thick transversal cryosections or decalcified, dehydrated, paraffinized, and cut into ~5-μm-thick transversal paraffin sections according to the standard procedures of the UKE Mouse Pathology Facility.

Immunohistochemistry was carried out as described previously (*8*) (except for CPEB3, which is described in detail below). Briefly, slices were incubated in 10% normal donkey serum in 0.1% Triton X-100 in PBS for 45 min, and the primary antibody (table S6) was incubated in PBS in a humidified slide staining system at 4°C overnight. All additional steps were carried out at room temperature. Fluorophore-labeled secondary antibody was incubated for 2.5 hours in a humidified chamber, and slices were embedded in ROTIMount FluorCare 4′,6-diamidino-2-phenylindole (DAPI) (Carl Roth, catalog no. HP20.1) and imaged with a confocal laser scanning microscope (LSM 700, Carl Zeiss).

CPEB3 staining was carried out as described previously with minor modifications (*56*, *57*). Briefly, paraffin-embedded cervical spinal cord slices were dewaxed, rehydrated, and processed by antigen retrieval in 10 mM sodium citrate buffer (pH 6.0) for 5 min in a pressure cooker. Slices were permeabilized with 0.1% Triton X-100 for 10 min and blocked with 5% BSA in PBS for 1 hour followed by incubation in Mouse-To-Mouse Blocking Reagent (ScyTek, catalog no. MTM008) for 1 hour and by incubation of the primary antibodies [affinity-purified polyclonal anti-rabbit CPEB3 (provided by Y.-S.H., Taiwan)] and monoclonal anti-mouse NeuN antibodies (Merck, catalog no. MAB377) at 1:20 and 1:250 dilution, respectively, in 1% BSA/PBS overnight at 4°C. After three washes with PBS, the slices were incubated with Alexa Fluor–conjugated secondary antibodies and Hoechst 33342 in the dark for 2 hours, washed with PBS thrice, and then mounted in ProLong Gold Antifade Mountant (Invitrogen, catalog no. P36934). Fluorescent images were acquired by confocal laser scanning microscopy (LSM 780, Carl Zeiss) at ×20 magnification with three Z-stacks at 1-μm intervals and constant exposure settings. CPEB3 fluorescence intensity within all NeuN-positive cells of the spinal cord ventral horn outflow tract was quantified by ImageJ. Analysis was standardized across conditions. At least three images were analyzed per animal, and the mean per animal was used for subsequent statistical comparison.

Histopathology was performed according to the standard procedures of the UKE Mouse Pathology Facility as described previously (*9*). Briefly, avidin-biotin complex technique with 3,3′-diaminobenzidine (brown stain) was used for immunolabeling and hematoxylin (blue color) as counterstaining. Images were analyzed with QuPath 0.30 software (https://qupath.github.io/). CD3- and IBA1-positive cells as well as Luxol Fast Blue staining area of the cervical spinal cord were analyzed using a customized counting mask. APP-positive deposits of the white matter tract and HuD-positive cells of the spinal cord ventral horn outflow tract were counted manually using the QuPath counting tool. All analysis conditions were standardized. At least three slices per animal were analyzed to calculate the mean per animal that was used for subsequent statistical comparisons. For HuD, a fire look-up table (LUT) was used. See table S6 for antibody specifications.

### Immunocytochemistry

Primary neuronal cells were fixed with 4% PFA in PBS for 15 min at room temperature and washed with PBS thrice. Cells were permeabilized and blocked in 10% normal donkey serum in 0.1% Triton X-100 in PBS for 45 min. The primary antibodies (table S6) were incubated for 60 min at room temperature. After three washes in PBS, cells were incubated with secondary antibodies diluted 1:500 for 90 min. After three washes in PBS, the coverslips were mounted in ROTIMount FluorCare DAPI (Carl Roth, catalog no. HP20.1) and imaged with a confocal laser scanning microscope (LSM 700, Carl Zeiss).

Nuclear pCREB immunocytochemistry was performed as described previously (*58*). Briefly, after fixation, cells were incubated in 2% glycine, 0.2% gelatine, 2% BSA, and 50 mM NH_4_Cl (pH 7.4) for 60 min and primary antibodies (table S6) were incubated for 60 min at 4°C. After three washes in PBS, samples were incubated with DAPI (Vectashield/Biozol, catalog no. CL-BCFA-211) 1:1000 in PBS for 10 min at room temperature and mounted in Mowiol 4-88 (Merck Chemicals). Images at ×60 magnification, Z-stacks at 0.3-μm intervals, and 207-nm pixel size were acquired with a confocal laser scanning microscope (Fluoview FV1000, Olympus). Per image, average pCREB immunofluorescence intensity was generated from three optical planes covering the nuclear region identified by DAPI staining in Fiji software. The values were normalized to the mean intensity of the control group per staining batch. For visualization of the quantified channel, a fire LUT was used.

### miRNA target gene luciferase assay

N2a cells (American Type Culture Collection) were kept in DMEM with high glucose, GlutaMAX supplement (Gibco, catalog no. 61965059), penicillin-streptomycin (50 U ml^−1^, Gibco, catalog no. 15070063), and 10% FBS (PAN Biotech, catalog no. P30-3306) in a cell culture incubator at 37°C, 5% CO_2_, and a relative humidity of 98% until approximately passage 25. After dissociation in 0.1% TrypLE (Gibco, catalog no. 12563029) supplemented with 1 mM EDTA (Invitrogen, catalog no. 15575020), 5 × 10^4^ cells/cm^2^ were seeded into PDL (50 μg ml^−1^) precoated polystyrene multiwell plates (Greiner). At a confluency of 80 to 85%, N2a cells were transfected with 150 ng of miTarget 3′UTR miRNA Target Clones (GeneCopoeia) with either a control 3′UTR (catalog no. CmiT000001-MT06) or the 3′UTR of *Cpeb3* (catalog no. MmiT079369) according to the manufacturer’s protocol (Lipofectamine 2000, Invitrogen, catalog no. 11668019). Assay vectors were cotransfected with 5 nM miRCURY LNA miRNA Mimics (Qiagen, catalog no. 339173) of either miR-92a (YM00470097-ADA) or a scrambled control miRNA (YM00479903-ADA). Luminescence was detected with the Dual-Glo Luciferase Assay System (Promega, catalog no. E2980) on Spark 10M multimode microplate reader (Tecan) 48 hours later.

### Single-molecule in situ hybridization

Human CNS tissue was fixed with 4% PFA, embedded in paraffin and cut into 2-μm-thin sections as described previously (*9*). Tissue sections were deparaffinized, and RNAscope Fluorescent Multiplex V2 [Advanced Cell Diagnostic (ACD), catalog no. 323100] was performed according to the manufacturer’s standard protocol for formalin-fixed paraffin-embedded tissue. The probes Hs-*CPEB3* (ACD, catalog no. 1106911-C1) and Hs-*SNAP25* (ACD, 518851-C3) were multiplexed on the same tissue. RNAscope human samples were scanned using the Pannoramic 250 FLASH II (3DHISTECH) Digital Slide Scanner at ×20 magnification. *CPEB3*-positive/*SNAP25*-positive neurons were quantified by a blinded experimenter using a custom-made script, which was based on Cognition Network Language (Definiens Cognition Network Technology; Definiens Developer XD software). All demographic data of human subjects and their tissue used are listed in table S7.

### Immunoblot

Mouse spinal cord or primary neuronal cells were lysed in radioimmunoprecipitation assay (RIPA) buffer containing 50 mM tris (pH 8), 150 mM NaCl, 5 mM EDTA (pH 8), 0.1% SDS, 1% NP-40, and 0.5% sodium deoxycholate in distilled water supplemented with protease inhibitor cocktail (cOmplete, Roche catalog no. 11697498001) and phosphatase inhibitor cocktail (PhosSTOP, Roche catalog no. 4906845001), homogenized with a tissue grinder pestle for microcentrifuge tubes, and incubated at 4°C for 30 min by end-over-end rotation. After centrifugation for 5 min at 3200*g* at 4°C, total protein concentration of the supernatant was determined with Pierce bicinchoninic acid protein assay kit (Thermo Fisher Scientific, catalog no. 23225). Supernatant was denatured in RIPA buffer containing NuPAGE LDS sample buffer (Invitrogen, catalog no. NP0007) and Bolt Sample Reducing Agent (Invitrogen, catalog no. B0009) for 10 min at 70°C. Fifteen micrograms of sample was loaded and run on Bolt 4 to 12%, bis-tris polyacrylamide gradient gels (Invitrogen, catalog no. NW04122BOX) in a Mini Gel Tank (Life Technologies) containing Bolt MOPS SDS running buffer (Invitrogen, catalog no. B000102). Proteins were then transferred to a methanol-activated polyvinylidene difluoride transfer membrane (Thermo Fisher Scientific, catalog no. 88518) in Bolt transfer buffer (Invitrogen, catalog no. BT00061) that was subsequently blocked in 5% BSA (Roth, catalog no. 8076.3) in Tris- buffered saline containg 0.1% Tween 20 (Sigma-Aldrich, catalog no. P1379) (TBS-T) and incubated together with the primary antibodies rabbit anti-CPEB3 1:300 (Abcam, catalog no. ab10883) and rabbit anti–β-actin 1:1000 (Cell Signaling Technology, catalog no. 4967) at 4°C overnight. The membrane was washed four times in TBS-T and incubated with 1:15,000 WesternSure Goat anti-rabbit horseradish peroxidase–conjugated secondary antibody (LI-COR Biosciences, catalog no. 926-80011) for 1 hour. The membrane was washed again, and the antibodies were detected by chemiluminescence using the WesternSure PREMIUM chemiluminescent substrate (LI-COR Biosciences, catalog no. 926-95000). Band intensities were finally recorded by using the ImageQuant LAS 4000 mini (GE Healthcare) and analyzed by Fiji software. CPEB3 band intensities were normalized to the respective β-actin values.

### Statistics

The detailed bioinformatic analyses are described in the respective sections of the article. Images were analyzed using Fiji (National Institutes of Health) or ImageJ software, respectively. Experimental data obtained by real-time cell viability assays were analyzed with a customized MATLAB script. Further experimental data were analyzed and visualized with the Prism9 software (GraphPad Software) for Mac. Unless otherwise stated, differences between two experimental groups were determined using an unpaired, two-tailed Student’s *t* test; differences between two paired experimental groups were determined using a two-tailed paired *t* test; differences between three or more experimental groups on a dependent variable were determined using one-way analysis of variance (ANOVA) corrected for multiple comparisons using Dunnett’s test; differences between two or more experimental groups on two variables were analyzed using two-way ANOVA corrected for multiple comparisons using Tukey’s test or Šídák’s test. Outlier analysis was performed with ROUT (*Q* = 10%). Correlations were calculated using one-tailed Pearson test. Statistical analysis of clinical EAE scores was performed applying two-tailed Mann-Whitney *U* test to the area under the curve (AUC) for each animal. Bar-dot plots are represented as mean ± SEM. Box plots are represented from the 25th to 75th percentile with median (middle line), and whiskers are drawn from the minimum to maximum value with all points shown. Violin plots indicate distribution of single values with median as middle line. Each dot/point represents the value derived from one biological replicate. Significant results are indicated by *P < 0.05, **P < 0.01, and ***P < 0.001.

### Study approval

Human tissue use for scientific purposes was in accordance with institutional ethical guidelines and approved by the ethics committee of the University of Geneva (Switzerland). Informed consent was obtained from subjects, in accordance with approval of the local ethical committee. Animal experimental procedures were in accordance with institutional guidelines and conformed to the requirements of the German Animal Welfare Act. Ethical approvals were obtained from the State Authority of Hamburg, Germany (no. 20/15, no. 122/17, and ORG 713 and ORG 1075).
