# Supplementary material for: MicroRNA-92a–CPEB3 axis protects neurons against inflammatory neurodegeneration
Source: Sci Adv. 2023 Nov 24;9(47):eadi6855. doi: 10.1126/sciadv.adi6855 (PMC10672163; doi:10.1126/sciadv.adi6855)
Supplement: Supplementary file 1 — Figs. S1 to S5 Legends for tables S1 to S7 [file sciadv.adi6855_sm.pdf]

Science Advances
AAAS

Supplementary Materials for

MicroRNA-92a–CPEB3 axis protects neurons against inflammatory neurodegeneration

Iris Winkler *et al.*

Corresponding author: Manuel A. Friese, manuel.friese@zmnh.uni-hamburg.de

Sci. Adv. **9**, eadi6855 (2023)
DOI: 10.1126/sciadv.adi6855

The PDF file includes:

Figs. S1 to S5
Legends for tables S1 to S7

Other Supplementary Material for this manuscript includes the following:

Tables S1 to S7

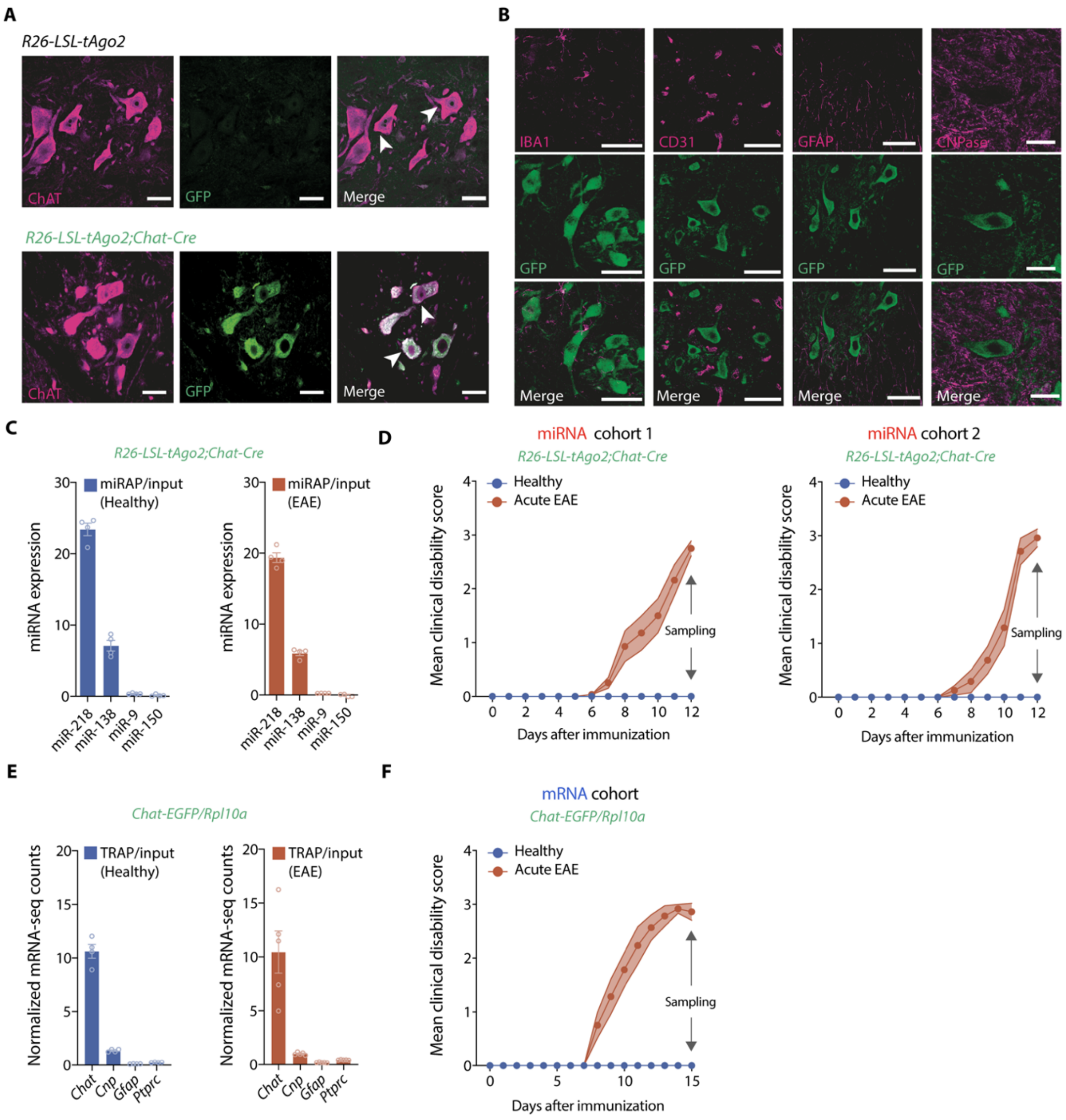

Fig. S1. Motor neuron-specific miRNA and mRNA purification of healthy and EAE mice. (**A**) GFP-tagged AGO2 (tAGO2) visualized by GFP counterstaining in ChAT-positive cells (white arrows) of Chat-Cre-positive and Chat-Cre-negative *R26-LSL-tAGO2* mouse spinal cord. Scale bars: 25 µm. (**B**) Immunohistochemical co-staining of tAGO2 and indicated cell type-specific marker proteins of the spinal cord (IBA1, microglia; CD31, endothelial cells; GFAP, astrocytes; CNPase, oligodendrocytes). Scale bars: 50 µm. (**C**) qRT-PCR of cell-specific miRNAs of spinal cord lysate (input) or motor neurons purified by GFP-AGO2 immunoprecipitation (miRAP) of healthy and acute EAE (day 12 post immunization) *R26-LSL-tAGO2;Chat-Cre* mice (miR-218, motor neuronal; miR-138, neuronal, miR-9 neural; miR-150 hematopoietic). miRNA expression is relative to miR-384 (identified as endogenous control for miRNA analyses in EAE; stable expression among healthy spinal cord, EAE spinal cord, healthy motor neuron and EAE motor neuron). (**D**) Disease course shown as mean clinical disability score of healthy and EAE *R26-LSL-tAGO2;Chat Cre* mice of two independent cohorts that were used for miRNA profiling. Spinal cords were collected at acute EAE. (**E**) mRNAs were either sequenced from whole spinal cord lysate (input) or motor neurons after GFP-RPL10A immunoprecipitation (TRAP) from healthy and acute EAE (day 15 post immunization) *Chat-EGFP/Rpl10a* mice. Bar plots show normalized RNA-seq counts of cell type-specific marker genes (*Chat*, motor neurons; *Cnp*, oligodendrocytes; *Gfap*, astrocytes; *Ptprc* (CD45), immune cells). (**F**) Disease course shown as mean clinical disability score of healthy and EAE *Chat-EGFP/Rpl10a* mice that were used for mRNA profiling.

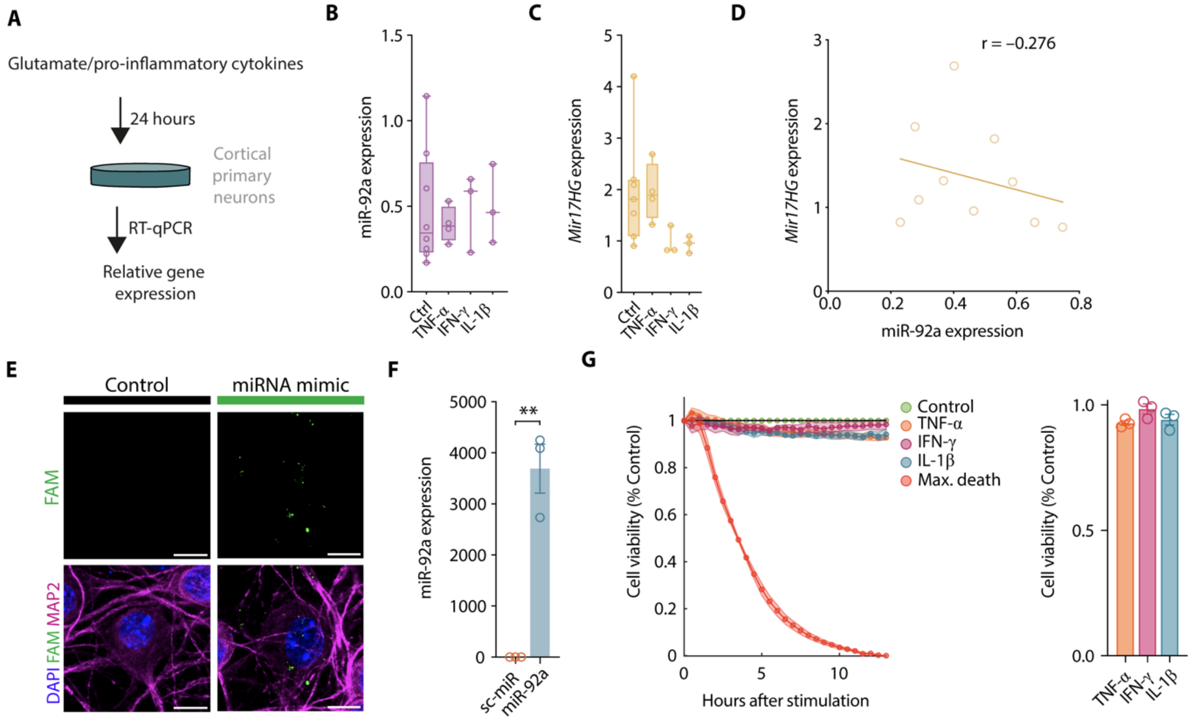

Fig. S2. miR-92a and *Mir17HG* expression are not influenced by pro-inflammatory cytokines in cortical neurons. (**A**) Scheme of experimental approach: primary neurons were stimulated with glutamate or pro-inflammatory cytokines for 24 hours and miRNA and gene expression tested by qRT-PCR. (**B**) qRT-PCR of miR-92a (relative to sno234) after stimulation of primary neurons with cytokines (100 ng ml^{-1}) for 24 hours. Ctrl, n = 8; tumor necrosis factor (TNF)-α, n = 5; interferon (IFN)-γ, n = 3; interleukin (IL)-1β, n = 5. ROUT outlier identification; One-way ANOVA, $F(3, 14) = 0.1282$, $P = 0.9418$. (**C**) qRT-PCR of *Mir17HG* (relative to *Tbp*) of neurons stimulated as in **B**. One-way ANOVA, $F(3, 13) = 2.040$, $P = 0.1580$. (**D**) Pearson correlation of relative miR-92a and *Mir17HG* expression assessed by qRT-PCR after exposure to different pro-inflammatory cytokines for 24 hours (number of XY pairs, 10; $P = 0.2199$). (**E**) Representative images of MAP2 immunocytochemistry of primary neurons transfected with either FAM-labeled miRNA mimics (25 nM) or transfection reagents only (control). Scale bars: 10 µm. (**F**) qRT-PCR on miR-92a (relative to sno234) of miR-92a mimic or sc-miR (15 nM) transfected primary neurons (n = 3). Student's t test, $P = 0.0015$. (**G**) Real-time viability assay of primary neurons stimulated with 100 ng ml^{-1} of the pro-inflammatory cytokines TNF-α, IFN-γ or IL-1β. Each well was normalized to its own baseline luminescence after 5 hours, to the unstimulated vehicle control (100% cell viability) and to the max death control (2 mM glutamate, 0% cell viability); n = 3.

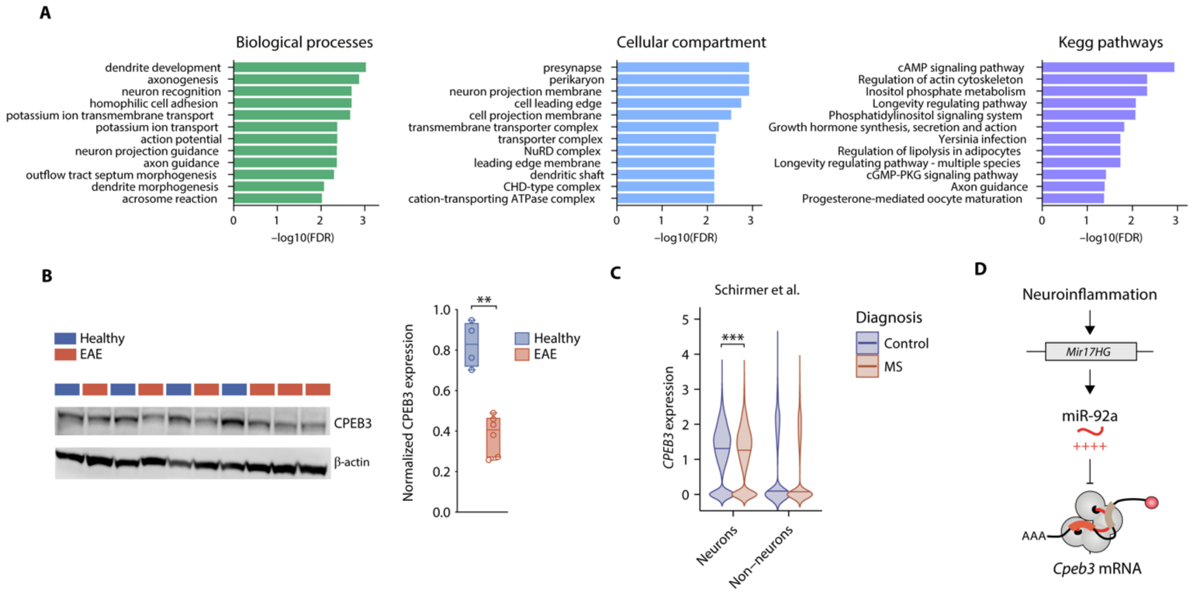

Fig. S3. miR-92a regulates neuronal pathways in neuroinflammation and CPEB3 is suppressed in EAE spinal cord and MS neurons. (**A**) Gene ontology and KEGG pathway over-representation analysis of identified miR-92a mRNA targets with resulting terms ordered by statistical significance (false discovery rate; FDR). (**B**) Immunoblotting against CPEB3 (normalized to β-actin) of spinal cord lysates of healthy (n = 4) and acute EAE (day 13 post immunization) mice (n = 6). One-tailed Mann-Whitney U test, $P = 0.0048$. (**C**) Normalized CPEB3 expression in human single nuclei data (*7*), control neurons n = 32,997, MS neurons n = 38,118, control non-neurons n = 19,212, MS non-neurons n = 56,430, Mann-Whitney U test against control, neurons $P < 0.0001$, non-neurons $P = 0.4150$. (**D**) Scheme summarizing the proposed neuronal miR-92a–*Cpeb3* axis.

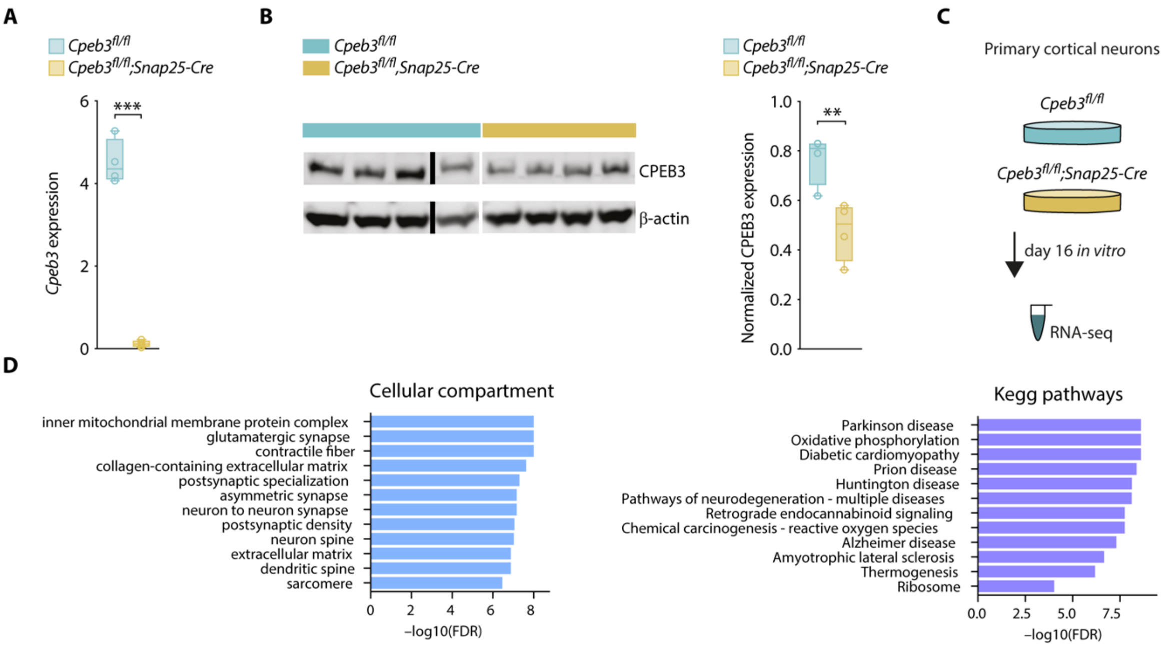

Fig. S4. Characterization of *Cpeb3$^{fl/fl}$;Snap25-Cre* primary neurons. (**A**) qRT-PCR of *Cpeb3* (relative to *Tbp*) in primary neurons of *Cpeb3$^{fl/fl}$;Snap25-Cre* mice and *Cpeb3$^{fl/fl}$* littermate controls. Student's *t*-test, $P < 0.0001$, $n = 4$. (**B**) Immunoblotting against CPEB3 (normalized to β-actin) of primary neurons derived from *Cpeb3$^{fl/fl}$;Snap25-Cre* and *Cpeb3$^{fl/fl}$* mice. Student's *t*-test, $P = 0.0098$, $n = 4$. Lanes were run on the same gel (noncontiguous lane indicated by black bar). (**C**) Scheme of experimental approach. (**D**) Gene ontology and KEGG pathway gene set enrichment analysis of primary neurons derived from *Cpeb3$^{fl/fl}$;Snap25-Cre* versus *Cpeb3$^{fl/fl}$* control mice with resulting terms ordered by statistical significance (false discovery rate; FDR).

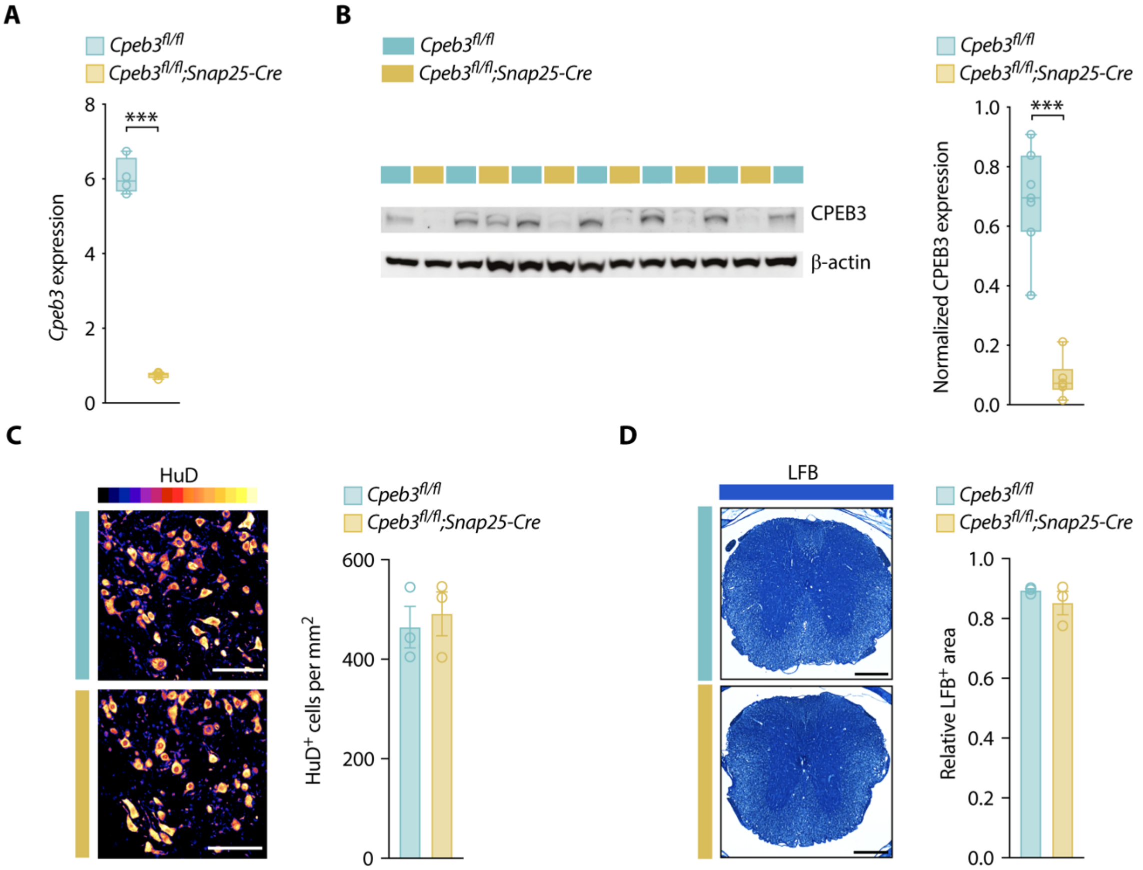

Fig. S5. Characterization of *Cpeb3$^{fl/fl}$;Snap25-Cre* mice. (**A**) qRT-PCR of *Cpeb3* (relative to *Tbp*) in spinal cord of *Cpeb3$^{fl/f}$;Snap25-Cre* and *Cpeb3$^{fl/fl}$* mice. Student's *t*-test, $P < 0.0001$, $n = 4$. (**B**) Immunoblotting against CPEB3 (normalized to β-actin) in spinal cord of *Cpeb3$^{fl/fl}$;Snap25-Cre* ($n = 6$) and *Cpeb3$^{fl/fl}$* mice ($n = 7$). Student's *t*-test, $P < 0.0001$. (**C**) Immunohistochemical staining and quantification of HuD$^+$ neurons in cervical spinal cord ventral horn of *Cpeb3$^{fl/fl}$;Snap25-Cre* versus *Cpeb3$^{fl/fl}$* control mice. Student's *t*-test, $P = 0.6803$, $n = 3$. Scale bar: 100 µm. (**D**) Staining of myelin with Luxol-Fast-Blue (LFB) in cervical spinal cords of *Cpeb3$^{fl/fl}$;Snap25-Cre* versus *Cpeb3$^{fl/fl}$* control mice. The LFB$^+$ area is normalized to the spinal cord area. Student's *t*-test, $P = 0.3468$, $n = 3$. Scale bar: 250 µm.

Other Supplementary Material for this manuscript includes:

Table S1. EAE miRNA sequencing
Table S2. EAE mRNA sequencing
Table S3. *Cpeb3*$^{-/-}$ neuron mRNA sequencing
Table S4. EAE miRNA–mRNA network
Table S5. qRT-PCR assays
Table S6. Antibodies
Table S7. Clinical data
